# Tumor lineage-specific immune response in brain metastatic disease: opportunities for targeted immunotherapy regimen?

**DOI:** 10.1186/s40478-023-01542-9

**Published:** 2023-04-15

**Authors:** Shiva Najjary, Johan M. Kros, Willem de Koning, Disha Vadgama, Karishma Lila, Janina Wolf, Dana A. M. Mustafa

**Affiliations:** 1 Department of Pathology and Clinical Bioinformatics, The Tumor Immuno-Pathology Laboratory, Erasmus University Medical Center, Dr. Molewaterplein 40, 3015 GD Rotterdam, The Netherlands; 2 Department of Pathology and Clinical Bioinformatics, Erasmus University Medical Center, Rotterdam, The Netherlands; 3 Present Address: Institute of Tissue Medicine and Pathology, University of Bern, Murtenstrasse 31, 3008 Bern, Switzerland

**Keywords:** Brain metastases, Lung adenocarcinoma, Breast cancer, Gene expression, Immune response, Immune infiltration

## Abstract

Metastases in the brain are the most severe and devastating complication of cancer. The incidence of brain metastasis is increasing. Therefore, the need of finding specific druggable targets for brain metastasis is demanding. The aim of this study was to compare the brain (immune) response to brain metastases of the most common tumor lineages, viz., lung adenocarcinoma and breast cancer. Targeted gene expression profiles of 11 brain metastasis of lung adenocarcinoma (BM-LUAD) were compared to 11 brain metastasis of breast cancer (BCBM) using NanoString nCounter PanCancer IO 360™ Panel. The most promising results were validated spatially using the novel GeoMx™ Digital Spatial Profiler (DSP) Technology. Additionally, Immune cell profiles and expression of drug targets were validated by multiplex immunohistochemistry. We found a more active immune response in BM-LUAD as compared to BCBM. In the BM-LUAD, 138 genes were upregulated as compared to BCBM (adj. *p* ≤ 0.05). Conversely, in BCBM 28 genes were upregulated (adj. *p* ≤ 0.05). Additionally, genes related to CD45 + cells, T cells, and cytotoxic T cells showed to be expressed higher in BM-LUAD compared to BCBM (adj. *p* = 0.01, adj. *p* = 0.023, adj. *p* = 0.023, respectively). The spatial quantification of the immune cells using the GeoMx DSP technique revealed the significantly higher quantification of CD14 and CD163 in tumor regions of BM-LUAD as compared to BCBM. Importantly, the immune checkpoint VISTA and IDO1 were identified as highly expressed in the BM-LUAD. Multiplex immunohistochemistry confirmed the finding and showed that VISTA is expressed mainly in BM-LUAD tumor cells, CD3 + cells, and to fewer levels in some microglial cells in BM-LUAD. This is the first report on differences in the brain immune response between metastatic tumors of different lineages. We found a far more extensive infiltration of immune cells in BM-LUAD as compared to BCBM. In addition, we found higher expression of VISTA and IDO1 in BM-LUAD. Taken together, targeted immune therapy should be considered to treat patients with BM-LUAD.

**Supplementary Information:**

The online version contains supplementary material available at 10.1186/s40478-023-01542-9.

## Introduction

The rise of brain metastases (BM) is the most severe and devastating complication of solid tumors, occurring in up to 50% of patients with metastatic cancer [1]. BM causes significant morbidity and negatively affects survival rates [2]. The incidence of BM has significantly risen in recent years due to better treatment modalities that resulted in longer survival times, opening wider windows for metastases to arise. Furthermore, the development of more sensitive diagnostic tools has increased the detection of BM [3]. The most common brain metastases arise from primary tumors in the lung, breast, melanoma, and colorectal cancer [4]. Most lung cancer brain metastases (LCBM) arise from lung adenocarcinomas (LUAD), rather early in the course of the disease [5, 6]. In contrast, breast cancer brain metastases (BCBM) are usually a late complication, of which the triple-negative (TNBC) and HER2 + breast cancer subtypes have the highest potency to migrate to the brain [7, 8]. The median survival of patients with LCBM is approximately 7–12 months and about 15 months in patients with BCBM [9, 10]. Despite the development of new therapies, novel targeted therapeutic agents have little effect on BM, partly due to the blood–brain barrier (BBB) obstacle for many drugs, and differences in sensitivity between primary tumors and their brain metastases [11, 12]. In order to develop targeted immunotherapies to improve treatment outcomes for patients with BM, a better understanding of the biological and immunological characteristics of BM and identification of the involved molecular mechanisms is of great importance.

After entering the brain, tumor cells face a specific and complex environment that is fundamentally different from the environment of the primary tumors in terms of cell composition, metabolism, and immune landscape [13]. The tumor cells, together with the cells in the brain, form a complex tumor microenvironment (TME) that maintains normal tissue homeostasis and hosts the immune response against metastatic tumor cells [14, 15]. Intercellular communication is a dynamic network of cytokines, chemokines, growth factors, and enzymes that remodel the extracellular matrix, leading to profound changes in the characteristics of the surrounding tissue [16]. Histopathological studies in various tumor types have shown infiltration of immune cells including macrophages, granulocytes, T lymphocytes, myeloid-derived suppressor cells (MDSCs), as well as cellular heterogeneity of the tumor niches [17–19]. Exploring the molecular differences between the primary tumor and the matched-paired brain metastasis has been performed previously [20–24]. In addition, the involvement of the immune system in cancer progression has been well established. However, little is known about the (immune) response of the brain toward various types of tumors [21, 25–27].

The aim of this study was to investigate the brain (immune) response toward different types of tumors. In addition, we aimed to discover targetable molecules in brain metastasis from various origins. Therefore, we compared cancer- and immune-related gene expression profiles in brain metastasis of lung adenocarcinoma (BM-LUAD) to that of BCBM. Our findings were confirmed spatially by using the novel GeoMx Digital Spatial Profiler (DSP) technique and by multiplex Immunohistochemistry. BM-LUAD showed more infiltration of the immune cells and higher expression of immune checkpoint targets than BCBM. The present findings suggest that specific immune therapy may benefit patients with brain metastasis of lung cancer.

## Materials and methods

### Tissue samples and clinical data

**Fig. 1:**
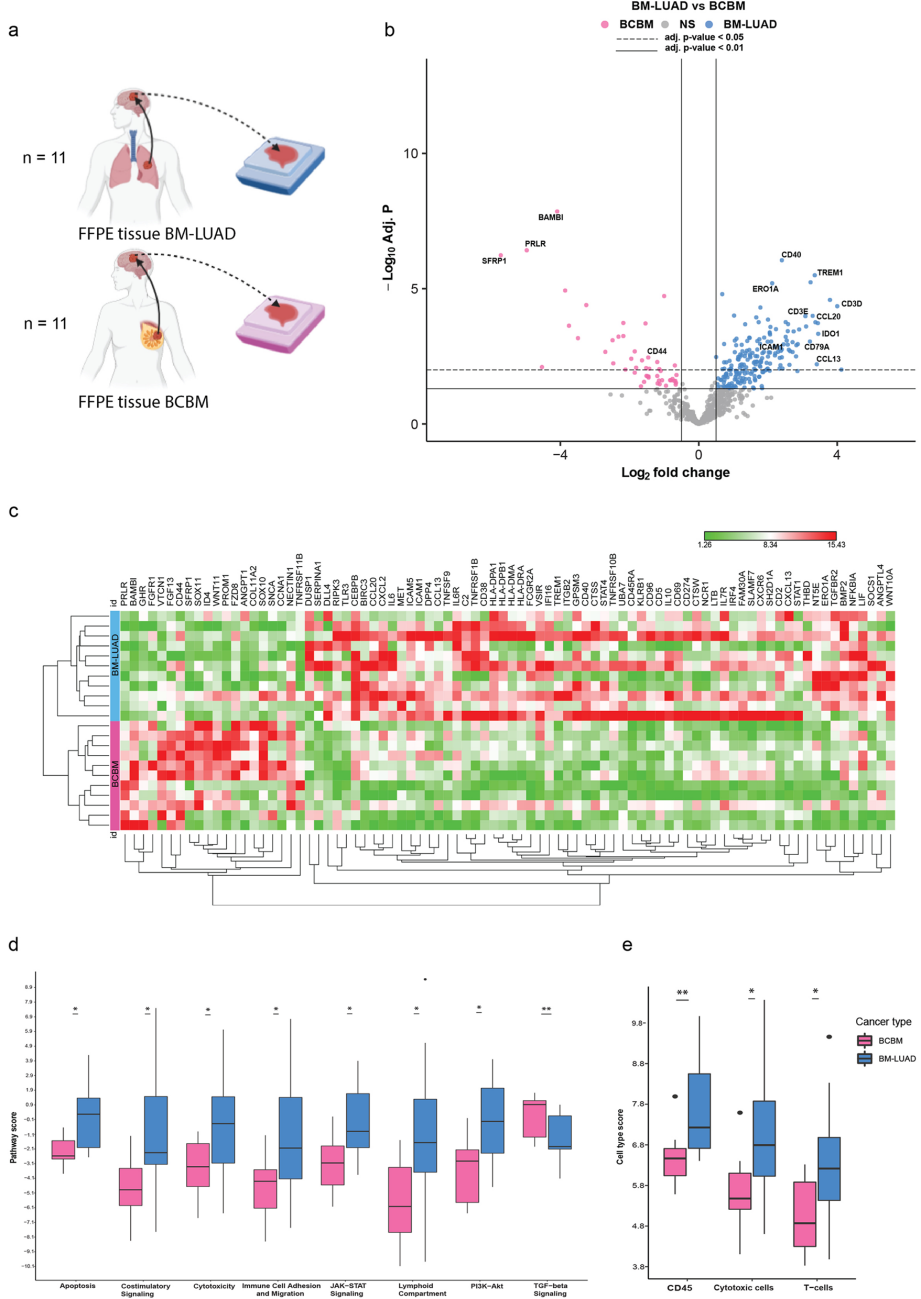
The Nanostring nSolver advanced analysis for the targeted gene expression, pathway signatures, and immune cell profiles between BM-LUAD and BCBM using nCounter PanCancer IO 360™ Panel. **a** Experimental set-up of targeted gene expression profiling (Nanostring nCounter PanCancer IO360 panel, n = 770 genes) and protein expression (GeoMx DSP) of BM-LUAD (n = 11) and BCBM (n = 11) from FFPE tissue samples. **b** Volcano plot indicating differentially expressed genes between BCBM and BM-LUAD (adj. *p* ≤ 0.05). **c** Heatmap of normalized differentially expressed genes between BCBM and BM-LUAD (absolute fold change ≥ 1; adj. *p* ≤ 0.05). The scaling of the heatmap is based on each gene. **d** Box plots of nCounter PanCancer IO 360™ biological signatures representing the set of pathway scores upregulated in each brain metastasis group (* adj. *p* ≤ 0.05; ** adj. *p* ≤ 0.01). **e** Box plots of immune cell scores acquired pursuant to the tumor location and showing higher levels of CD45 + cells notably T cells and cytotoxic cells in BM-LUAD (* adj. *p* ≤ 0.05; ** adj. *p* ≤ 0.01)

The unique cohort of twenty-two Formalin-fix, paraffin-embedded (FFPE) tissue samples of brain metastasis from lung adenocarcinoma (n = 11) and breast cancer (n = 11) were collected (Fig. 1a). The clinical characteristics of patients are summarized in Table 1. The median age at diagnosis of the patients with BM-LUAD was 64 years and of the patients with BCBM 48 years. The ER, PR, and her2neu status of the primary breast cancers and their matched brain metastases are shown in (Table 1). None of the patients in either group received therapy or corticosteroids 6–12 months prior to brain metastasis surgery. Treatments for BM after the surgical removal of the brain metastasis were radiotherapy, chemotherapy, stereotactic radiotherapy (SRT), and whole brain radiotherapy (WBRT). The histopathology of primary breast cancer and their matched BM showed some discordancy (Table 1). That was mainly observed in the primary triple-negative breast cancer (TNBC) subtype. While 5 (out of 11) primary samples were TNBC, only 2 (out of 11) remained TNBC when developing metastasis to the brain. This study was approved by the Medical Ethics Committee of the Erasmus Medical Center, Rotterdam, the Netherlands (MEC 02·953 & MEC-2020–0732), and was conducted in adherence to the Code of Conduct of the Federation of Medical Scientific Societies in the Netherlands**.**

**Table 1:** Clinical characteristics of brain metastasis from lung and breast cancer

Characteristics	No	%
Total samples	22	100
Cancer type		
Brain metastasis from lung adenocarcinoma	11	50
Brain metastasis from breast cancer	11	50
**Lung cancer**		
Median age at diagnosis, years (range)	64 (46–74)	
Sex		
Male	6	54.5
Female	5	45.5
Histology		
Adenocarcinoma	11	100
Smoking status		
Never smoking	1	9.1
Former smoker	5	45.5
Smoking	4	36.4
Unknown	1	9.1
Treatment after surgery of BM		
No treatment	1	9.1
Radiotherapy	6	54.4
SRT	1	9.1
WBRT	2	18.2
Other	1	9.1
**Breast cancer**		
Median age at diagnosis, years (range)	48 (36–74)	
Histology of primary tumor		
ER/PR +	2	18.2
ER/HER2 +	1	9.1
HER2 +	1	9.1
TNBC	5	45.5
Unknown	2	18.2
Histology of matched-BM		
ER +	3	16.7
PR +	2	11.1
ER/PR +	1	5.6
HER2 +	2	11.1
TNBC	2	11.1
Unknown	1	5.6
Treatment after surgery of BM		
Radiotherapy	5	45.5
Chemotherapy	2	18.2
Radiotherapy & Chemotherapy	2	18.2
Other	2	18.2

ER: estrogen receptor; PR: progesterone receptor; HER2: human epidermal growth factor 2; TNBC: triple-negative breast cancer; SRT: Stereotactic radiotherapy; WBRT: Whole brain radiotherapy

### RNA extraction and quality control

RNA extraction was performed as explained previously [28]. In short, tissue sections of 5 μm were stained with hematoxylin and eosin (H&E) and examined by a pathologist. Total RNA was extracted from 10–12 sections of 10 μm thickness using the RNeasy FFPE kit (Qiagen, Hilden, Germany) according to the manufacturer’s instructions. RNA was stored in RNase/DNase-free water at -80 °C. The quality and quantity of extracted RNA were assessed by Agilent 2100 Bioanalyzer (Santa Clara, CA, USA). RNA degradation was calculated using percentages of fragments of 300–4000 nucleotides.

### Targeted gene expression analysis using nanostring® technology

Gene expression was measured using the PanCancer IO 360™ Panel (Nanostring Technologies, Seattle, WA, United States), consisting of 770 genes related to cancer biology, the microenvironment, the immune response, and housekeeping genes as described previously [29]. In short, 300 ng of good quality RNA, with a maximum of 7 μL was used for hybridization with the panel probes for 17 h at 65 °C using a SimpliAmp Thermal Cycler (Applied Biosystems, Foster City, CA, USA). Cleaning of the extra unannealed probes was performed using the nCounter FLEX system and the expression of genes was calculated by scanning 490 fields-of-view (FOV).

Expression data were uploaded to the nSolver software (4.0), and data analysis was done using the Advanced Analysis module (2.0). The most stable housekeeping genes (Additional file 1: Table S1) were used to normalize the raw expression data using the geNorm algorithm, and the background threshold was set as the mean of negative controls plus 2 standard deviations.

### Immune cell deconvolution and pathway analysis

To characterize the relative abundance of immune cells, immune cell-specific gene markers were chosen by calculating the pairwise similarity between all pairs of candidate marker genes (n = 61) that were above the background detection limit [30]. Gene pairs that showed pairwise similarity of > 0.6 were selected to identify immune cells. The cell type score is the average of log-transformed expression values of marker genes which were used to compare the relative abundance of immune cells between BM-LUAD and BCBM. Gene Set Analysis (GSA), embedded within the nSolver software, was used to evaluate the differences at the pathways level. Pathway scores were calculated using the average expression of genes that were associated with a designated pathway. The significant changes for the abundance of immune cells and for pathway scores were calculated using the *t*-test between BM-LUAD and BCBM.

### GeoMx digital spatial profiler (DSP)

The spatial expression of immune-related targets was performed as described previously [31]. In short, 5 um of brain metastasis FFPE was used for this experiment. Various fluorescent-labeled antibodies were used as morphological markers. At the same time, a cocktail of 79 antibodies including the immune-related targets, housekeeping proteins, and negative controls (Additional file 1: Table S2). Two regions of interest (ROIs) were selected in each sample: tumor-rich ROI and immune-rich ROI. Each ROI Antibodies counting was achieved following the manufacturer’s instructions (NanoString Technologies, Seattle, WA, USA). The protein expression data were normalized to two housekeeping proteins (Histone H3, S6) and corrected for the background by subtracting the expression of the negative control Ms.IgG2a from data expression in every ROI separately. The Linear Mixed Model (LMM) was sued to calculate the significant differences between the 2 groups, and proteins were considered significantly expressed when (adj. *p* < 0.5).

### Immunohistochemistry

All 22 tissue samples were used to validate the significantly expressed proteins by performing multiplex Immunohistochemistry (IHC). For the immune cell types, tissue sections of 5 μm were stained with CD163, CD14, PanCk antibodies and with syto13 (DNA) nuclear stating and scanned using the GeoMx DSP instrument. Additionally, all 22 samples were used for the conventional IHC to validate the expression of VISTA and IDO1 using Alkaline phosphatase, according to the manufacturer's standard protocol. The stained slides were scored and interpreted by a pathologist. VISTA was shown to be significantly highly expressed in the BM-LUAD group. In order to identify the specific cells that express VISTA in BM-LUAD samples, we performed multiplex immunofluorescence (IF) staining using 2 independent BM-LUAD samples by following the automated protocol using the Ventana Benchmark Discovery (Ventana Medical Systems Inc). The process of staining was carried out using a previously published method [31]. A summary of all antibodies is shown in Additional file 1: Table S3.

### Statistical analysis

The differential expression pattern of genes between BM-LUAD and BCBM was analyzed using a simplified negative binomial model and Benjamin-Hochberg procedures were applied to correct for multiple testing. Differences in pathway scores and cell type scores were assessed with the Mann–Whitney U test. All statistical analyses were carried out using R statistical software, version 4.0.1. The *P*-values were two-sided, and a *P*-value ≤ 0.05 was considered statistically significant. The protein expression differences between BM-LUAD and BCBM were assessed using GeoMx DSP Analysis software, version 2.4.0.147. Benjamin-Hochberg method and the linear mixed model were applied to account for multiple observations within a given sample. Heatmaps were generated with Log2-normalized data of significantly expressed targets (adj. *p* ≤ 0.05). The Heatmap of DEGs was generated based on |Log2Fold change|> 1, BH < 0.05, and outliers were removed by using Tukey’s rule [32]. The web-based tool Morpheus by Broad Institute (RRID: SCR_017386) was used for the visualization of data as a heatmap.

## Results

### Differential targeted gene expression patterns between BM-LUAD and BCBM

The expression of 51/770 genes was below the detection limit in BM-LUAD and BCBM. A total of 166 genes were identified as DEGs (adj. *p* ≤ 0.05, Fig. 1b), of which 138 were upregulated in BM-LUAD (adj. *p* ≤ 0.05) and 28 were upregulated in BCBM (adj. *p* ≤ 0.05). The most significant differentially upregulated genes considering the lowest adj. *p*-value were present in the BM-LUAD and included CD40, TREM1, and ERO1A. The most significant upregulated genes based on the lowest adj. *p*-value in BCBM included BAMBI, PRLR, and SFRP1. In BM-LUAD higher expression (adj. *p* ≤ 0.05) was present of the chemokines CCL5, CCL13, CCL20, CXCL2, CXCL9, and CXCL13; the cellular adhesion molecule ICAM1; the T-cell markers CD3D and CD3E; the B cell marker CD79A and the monocytes marker CD40. Conversely, only CD44 (adj. *p* ≤ 0.05) was overexpressed in BCBM. Importantly, in BM-LUAD the immune checkpoint inhibitors VSIR and IDO1 were upregulated (adj. *p* ≤ 0.05; Fig. 1c).

### More active immune response and higher immune cell expression in BM-LUAD than BCBM

Eight pathway scores out of 25 signaling pathways were significantly different between BM-LUAD and BCBM (adj. *p* ≤ 0.05). In BM-LUAD there was enrichment for genes related to apoptosis (adj. *p* = 0.013); cytotoxicity (*p* = 0.034); co-stimulatory signaling (adj. *p* = 0.023); immune cell adhesion and migration (adj. *p* = 0.047); lymphoid compartment (adj. *p* = 0.028); JAK-STAT signaling (adj. *p* = 0.013) and PI3K-AKT signaling (adj. *p* = 0.016) (Fig. 1d). The BCBM were enriched only for genes related to TGF-beta signaling (adj. *p* = 0.01) (Fig. 1d). Using the pairwise similarity method to identify immune cell types in the samples resulted in identifying 13 immune cells types. The overall immune cell expression was higher in BM-LUAD than in BCBM. In general, there were more (CD45 +) immune cells present in the BM-LUAD samples (adj. *p* = 0.01). In particular, T cells and cytotoxic T cells were found to be relatively more abundant in BM-LUAD compared to BCBM (adj. *p* = 0.023, adj. *p* = 0.023, resp.) (Fig. 1e). However, B cells, DC, CD8 T cells, Exhausted CD8 T cells, macrophages, mast cells, NK cells, neutrophils were found to be equally abundant in both groups.

### Spatial biology confirmed the higher protein expression of immune-related targets in BM-LUAD

**Fig. 2:**
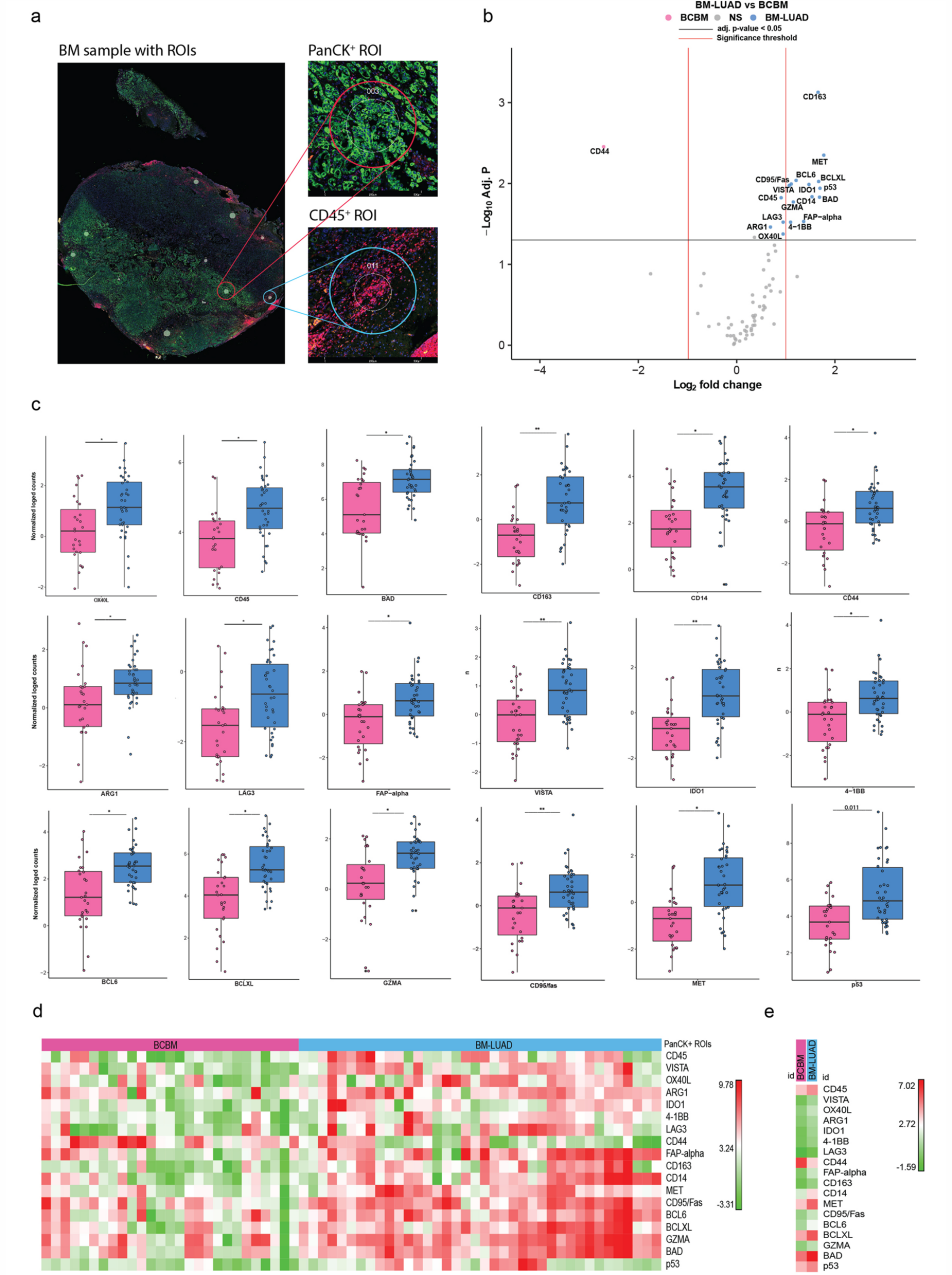
Digital spatial profiling of BM-LUAD and BCBM. **a** Image of a brain metastasis tissue sample used for DSP analysis and stained with morphological markers DNA (nucleus, blue), PanCK (tumor, green), and CD45 (immune cells, pink). **b** Volcano plot of differential expression of proteins in PanCK + tumor regions between BCBM and BM-LUAD (absolute fold change ≥ 1 and adj. *p* ≤ 0.05). **c** Box plots of protein targets in tumor cells containing compartments between BCBM and BM-LUAD. Each dot is presenting one PanCK + ROI in a BM sample, each ROI was measured in three replicates in all the samples. **d** Heatmap of normalized protein expression in PanCK + tumor ROIs between BCBM and BM-LUAD. The scaling of the heatmap is based on each target. **e** Heatmap of median normalized protein expression in tumor cell containing compartments (PanCK + ROIs) between BCBM and BM-LUAD

**Fig. 3:**
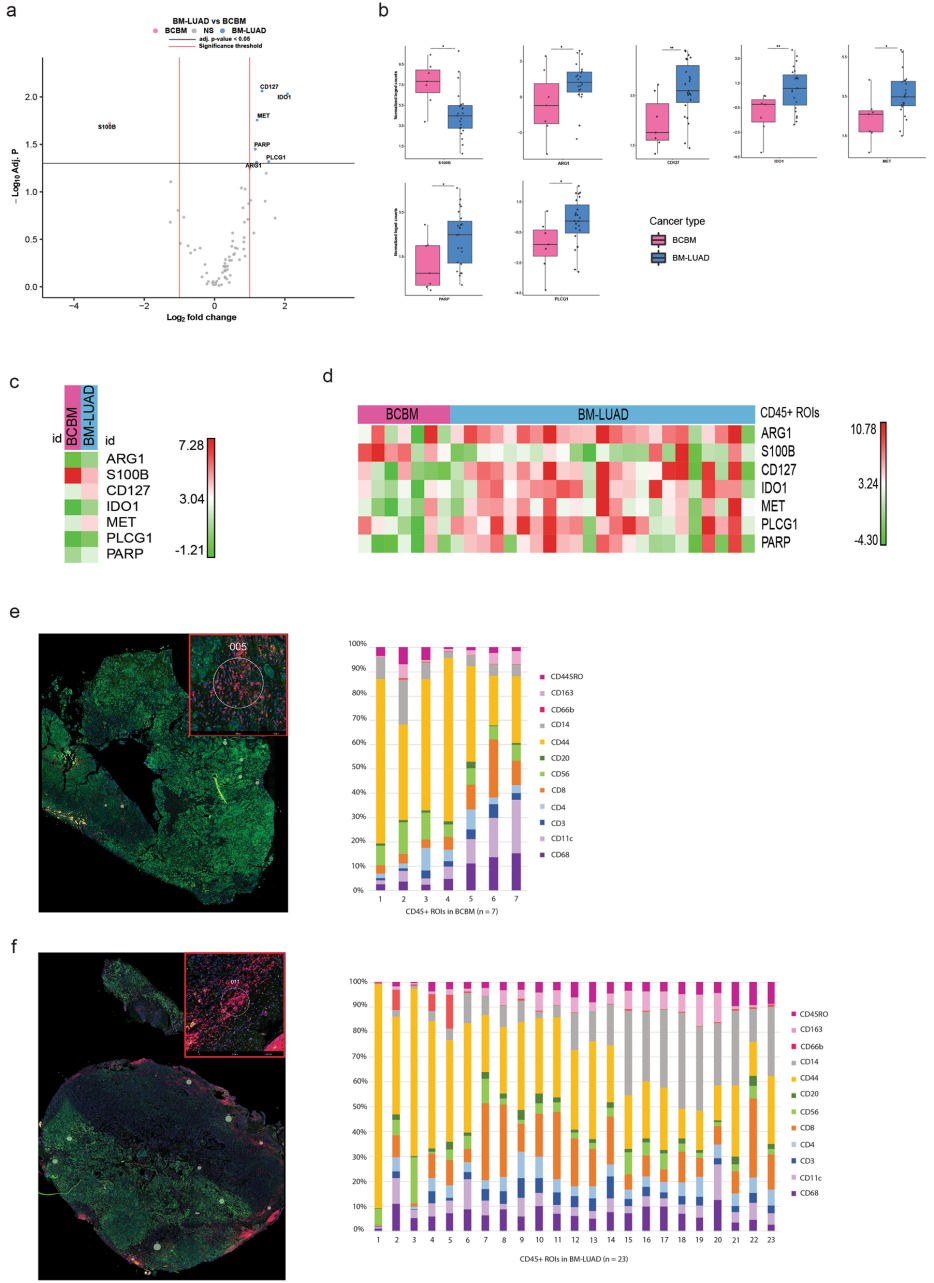
Digital spatial profiling of BM-LUAD and BCBM. **a** Volcano plot of differential expression of proteins in immune infiltrate regions (CD45 + area) between BCBM and BM-LUAD (absolute fold change ≥ 1 and adj. *p* ≤ 0.05). **b** Box plots of protein targets in immune infiltrate compartments between BCBM and BM-LUAD. Each dot is presenting one CD45 + ROI in a BM sample. (* adj. *p* ≤ 0.05; ** adj. *p* ≤ 0.01). **c** Heatmap of median normalized protein expression in CD45 + ROIs between BCBM and BM-LUAD. **d** Heatmap of normalized protein expression in CD45 + ROIs between BCBM and BM-LUAD. The scaling of the heatmap is based on each target. **(e)** Image of a CD45 + ROI in BCBM sample. The bar graph shows the distribution of immune cell types in seven immune ROIs from BCBM. Image of a CD45 + ROI in BM-LUAD sample. **f** The bar graph shows the distribution of immune cell types in twenty-three immune ROIs from BM-LUAD

The expressional differences were validated by comparing protein expression in different ROIs within the same tissue sample, namely tumor-rich and immune-rich ROIs (Fig. 2a). The spatial multiplex-protein measurements were carried out in 9/11 BCBM and 11/11 BM-LUAD samples of the discovery set (the tumor compartment was lost in 2 BCBM samples due to the amount of samples that was used for RNA extraction). Most of the examined targets tailored in the DSP panel were found to be higher expressed in BM-LUAD as compared to BCBM (Fig. 2b). Confirming the gene expression results, CD45 expression was found to be significantly higher in BM-LUAD samples (adj. *p* ≤ 0.05). However, in addition to confirming the results, spatial measurements revealed that the significant expression of CD45 in BM-LUAD was found only in tumor ROIs. Tumor ROIs in BM-LUAD expressed higher expressions of CD14, CD163, GZMA, BCL-6, BAD, BCLXL, 4-1BB, VISTA, and IDO1. The only protein that was significantly higher in the BCBM group is CD44 (adj. *p* ≤ 0.05), confirming the gene expression results obtained earlier (Fig. 2b–e). In addition, the expression of targets including tumor suppressor P53 and MET tyrosine-protein kinase were higher in BM-LUAD (Fig. 2b–e). Importantly, while selecting immune-rich ROIs in both groups, we discovered that those areas were much easier to find in BM-LUAD (n = 25) as compared to BCBM (n = 7) (Fig. 3d). Despite the higher immune-rich ROIs in the BM-LUAD as compared to BCBM, protein expression (Fig. 3a–d) and the composition of the immune cells was very similar between the BM-LUAD and BCBM (Fig. 3e and f).

### M2 macrophages polarization and drug targets were higher in BM-LUAD compared to BCBM

**Fig. 4:**
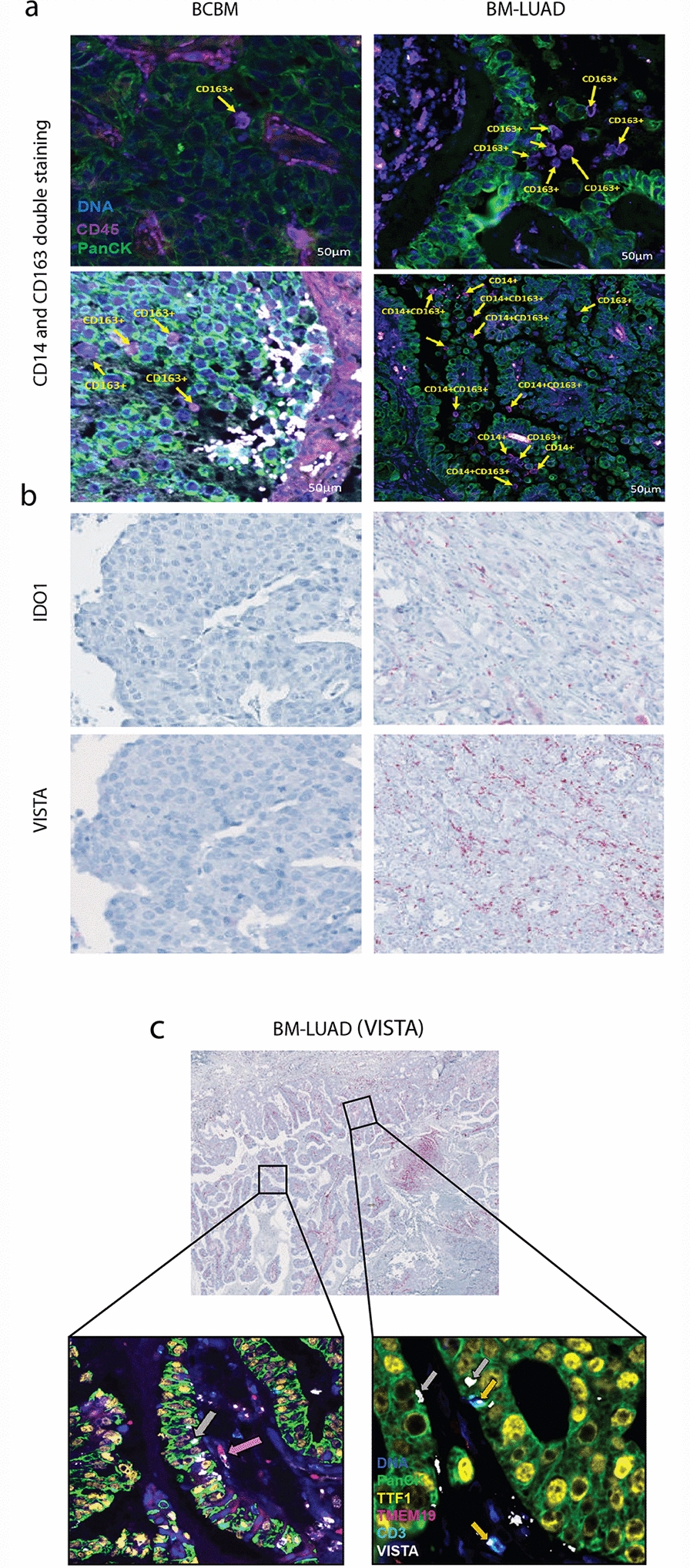
Digital spatial profiling of BM-LUAD and BCBM. **a** IHC double staining by DSP for immune cell infiltration (CD163 + M2 macrophages and CD14 + monocytes) in BCBM and BM-LUAD. **b** IHC staining for the expression of therapeutic targets VISTA and IDO1 in BCBM and BM-LUAD. **c** Multicolor fluorescence localizing expression of VISTA in BM-LUAD. The grey arrow points to the expression of VISTA in tumor cells; the pink arrow points VISTA expression in microglial cells; the orange arrow points VISTA expression in immune cells (Yellow: TTF1 [Thyroid transcription factor], Aqua: CD3, Red: TMEM19 [Transmembrane Protein 19], White: VISTA, Green: PanCK)

The higher expression of CD14 and CD163 in BM-LUAD was confirmed by fluorescent IHC. The expression of CD14 + and CD163 + cells was found to be in between tumor cells (Fig. 4a). The cells that express both proteins at the same time were found only in BM-LUAD samples. These results were obtained by using the GeoMx DSP technique but not by bulk RNA gene expression, highlighting the high sensitivity level of the GeoMx DSP. IHC revealed the higher expression of VISTA in all 11 BM-LUAD samples used in our study (Additional file 2: Fig. S1 and Fig. S2). Only 7 samples (out of 9 BCBM) showed some positivity of VISTA expression (Additional file 2: Fig. S1 and Fig. S2). On the other hand, IDO1 was expressed to a lower level in both groups and confirmed to be higher in BM-LUAD (Additional file 2: Fig. S1 and Fig. S2). The IHC results confirmed the same direction of expression obtained by gene expression profiles and by the spatial protein profiles. By using multiplex IF IHC, we found that the higher expression of VISTA was not confounded in tumor cells only. The expression of VISTA also co-localized with CD3 T cells and to a lower level in TMEM19 microglia cells. Additionally, screening for the expression of drug targets in brain metastasis of both cancers revealed a high level of VISTA and IDO1 in BM-LUAD, with relatively higher expression of VISTA compared to IDO1 (Fig. 4b). Using the 5-plex immunofluorescence staining, we observed the expression of VISTA in tumor cells, CD3 + cells, and at a lower level on microglial cells in BM-LUAD (Fig. 4c).

## Discussion

In the present study, we compared the brain response to metastasis of the lung (BM-LUAD) and breast (BCBM) and identified 166 differentially expressed genes. Most of the DE genes and the pathways associated with them were higher in BM-LUAD, suggesting that the brain responds differently to the different types of cancerous cells. About 55% of the DE genes were immune-related and found to be higher in the LUADBM group. By confirming the results spatially, we found that the immune infiltration is located in between tumor cells of LUAD, but not in BC. The infiltration is composed of T cells (CD3), Cytotoxic T cells (GZMA), and myeloid-derived cells (CD14, CD163). In addition, We found that the brain enables higher infiltration of immune cells when receiving LUAD cells compared to BC cells. That was reflected by the number of immune-rich ROIs that were hardly found and selected in the BC samples compared to LUAD samples. despite the significant difference in the immune-rich areas, the cellular composition of the immune infiltrates appeared to be similar. In addition, genes coding for chemokines, immune checkpoints (e.g. VSIR, IDO1), and the leukocyte adhesion molecule ICAM1 were overexpressed in the BM-LUAD. Furthermore, levels of the immune regulatory receptors VISTA and IDO1 were significantly higher in BM-LUAD as compared to those in BCBM. Pathway analysis revealed a more active immune response in the BM-LUAD compared to BCBM. The findings were validated by DSP and multiplex IHC and it appeared that in BM-LUAD the immune cells merged more often in between the tumor cells of BM-LUAD, hinting at a more intense immune interaction than observed in BCBM.

Studies on the identification of immunological features between brain metastases from different types of cancers are limited. Kudo et al. showed increased infiltration of M2 macrophages in brain metastasis from paired NSCLC samples by comparative immune gene profiling analysis [21]. In a recent study on human NSCLC, Zhang et al. showed increased expression of CD163 M2 macrophages in the tumor brain microenvironment and linked this finding with a significant promotion of neo-angiogenesis [33]. Berghoff et al. found differences in the infiltration of microglia and M2 macrophages between brain metastasis from NSCLC and melanoma [34]. In the present comprehensive analysis, we also found more infiltration of M2 macrophages in BM-LUAD as compared to BCBM. However, the increased numbers of M2 macrophages were accompanied by an overall higher immune activity in the TME of BM-LUAD. The finding of the significant overexpression of the immune checkpoint proteins VISTA and IDO1 in the BM-LUAD compared with the BCBM tumor-rich compartments of the brain metastases should shape lineage-specific brain metastasis therapeutic approaches. The expression of VISTA so far reported in NSCLC (primary tumors) is in line with our findings [35–38]. VISTA is an immune regulatory receptor predominantly expressed by myeloid cells with antigen-presenting properties like microglial cells [39]. We found that the expression of VISTA is not confined to microglia, but is also present in tumor cells and T lymphocytes, reflecting the situation in primary lung cancer [35]. High expression of VISTA has been associated with worse overall survival (OS) in various cancers [37]. The specificity for NSCLC may be attributed to lymphocyte enrichment in the TME of NSCLC as compared to that of other cancers [40–42]. Since VISTA is a ligand in antigen-expressing cells but is also present in T cells, investigations on the interference with VISTA in LCBM are necessary to discover the effects of being used as a target for immune therapy.

IDO1 is also an immune response-modulating molecule that we found significantly overexpressed in the LCBM in the present study. IDO1 is a suppressor of the immune response and a rate-limiting enzyme in tryptophan catabolism. Expression of IDO1 is induced by interferon-gamma and therefore related to the presence of T cells. IDO1 helps cancer cells to escape the immune response by tryptophan depletion from the TME and by producing the catabolic products of tryptophan degradation that are toxic to T cells and NK cells. High expression of IDO1 has been observed in various malignant tumors including lung cancers and its overexpression is associated with unfavorable clinical outcomes [43–47]. Zhao et al*.* found that IDO1 was highly expressed at the later stage of lung cancer suggesting that it may have a role in tumor progression [48]. So far, therapeutic interference with the IDO1 pathway has yielded a wide range of responses in cancers of various lineages [49, 50]. The present finding of predominant expression of IDO1 in BM-LUAD, not in BCBM, should be taken into consideration when developing combines therapeutic strategies with the involvement of IDO1.

In conclusion, this comprehensive comparison of the immune response in BM-LUAD and BCBM revealed that BM-LUAD are, in contrast with BCBM, highly immunogenic tumors with larger numbers of immune cells, larger numbers of activated pathways, and significant expression of the immune checkpoint molecules VISTA and IDO1. Therefore, BM-LUAD is an immunological “hot” tumor as opposed to BCBM, and treatment strategies should be developed accordingly.

## Supplementary Information

**Additional file 1. **Table S1. The list of housekeeping genes used for the normalization of genes. Table S2. List of (A) morphological markers, and (B) antibodies included in the core panel and module used in DSP. Table S3. Details of antibodies used in multiplex immunofluorescence staining.

**Additional file 2. Fig. S1** **Heterogeneity of VISTA and IDO1 expression in immunohistochemistry (IHC)-stained brain metastases tissues.** A. Expression of IDO1 and VISTA in IHC-stained BCBM. The numbers refers to a number of that sample (total samples = 9). B. Expression of IDO1 and VISTA in IHC-stained BM-LUAD. The numbers refers to a number of that sample (total samples = 11). **Fig. S2** **Comparison of immune checkpoint expressions in high expressed ROIs and low expressed ROIs of IHC-stained brain metastases tissues.** A. Expression of IDO1 and VISTA in IHC-stained BCBM in high expressed - and low expressed ROI. NLC stands for the normalized logged counts. The numbers next to the BCBM, refer to a number of that sample. B. Expression of IDO1 and VISTA in IHC-stained BM-LUAD in high expressed - and low expressed ROI. NLC stands for the normalized logged counts. The numbers next to the BM-LUAD, refer to a number of that sample.

## Data Availability

The datasets used and analyzed in the current study are available upon reasonable request from the corresponding author. Additional data included in the study are available in supplementary materials.
