# Supplementary material for: Tumor lineage-specific immune response in brain metastatic disease: opportunities for targeted immunotherapy regimen?
Source: Acta Neuropathol Commun. 2023 Apr 15;11:64. doi: 10.1186/s40478-023-01542-9 (PMC10105417; doi:10.1186/s40478-023-01542-9)
Supplement: Supplementary file 1 — Additional file 1. Table S1. The list of housekeeping genes used for the normalization of genes. Table S2. List of (A) morphological markers, and (B) antibodies included in the core panel and module used in DSP. Table S3. Details of antibodies used in multiplex immunofluorescence staining. [file 40478_2023_1542_MOESM1_ESM.docx]

**Table S1. The list of housekeeping genes used for the normalization of genes.**

| **N** | **Gene symbol** | **Gene name** | **SD** |
| --- | --- | --- | --- |
| 1 | UBB | Ubiquitin B | 0.495 |
| 2 | PUM1 | Pumilio RNA Binding Family Member 1 | 0.319 |
| 3 | TLK2 | Tousled Like Kinase 2 | 0.551 |
| 4 | DNAJC14 | DnaJ Heat Shock Protein Family (Hsp40) Member C14 | 0.628 |
| 5 | SF3A1 | Splicing Factor 3a Subunit 1 | 0.39 |
| 6 | POLR2A | RNA Polymerase II Subunit A | 0.575 |
| 7 | SDHA | Succinate Dehydrogenase Complex Flavoprotein Subunit A | 0.389 |
| 8 | STK11IP | Serine/Threonine Kinase 11 Interacting Protein | 0.594 |
| 9 | NRDE2 | NRDE-2, Necessary for RNA Interference, Domain Containing | 0.506 |
| 10 | TBP | TATA-Box Binding Protein | 0.467 |
| 11 | TBC1D10B | TBC1 Domain Family Member 10B | 0.376 |
| 12 | PSMC4 | Proteasome 26S Subunit, ATPase 4 | 0.604 |
| 13 | ABCF1 | ATP Binding Cassette Subfamily F Member 1 | 0.578 |
| 14 | TMUB2 | Transmembrane And Ubiquitin Like Domain Containing 2 | 0.486 |
| 15 | MRPL19 | Mitochondrial Ribosomal Protein L19 | 0.394 |
| 16 | ERCC3 | ERCC Excision Repair 3, TFIIH Core Complex Helicase Subunit | 0.358 |

*Note.* Order of HKGs is selected by geNorm algorithm of nSolver; SD: standard deviation after normalization.

**Table S2.** List of *(A) morphological markers, and (B) antibodies included in the core panel and module used in DSP.*

| A |  |  |  |  |  |  |
| --- | --- | --- | --- | --- | --- | --- |
| Morphological markers | **Cells / Compartment** |  |  |  |  |  |
| Syto13 | Nuclei / DNA |  |  |  |  |  |
| PanCK | Epithelial cells / Tumor |  |  |  |  |  |
| CD45 | Leukocytes / Immune cell |  |  |  |  |  |
| B |  |  |  |  |  |  |
| Immune Cell Profiling Core | **IO Drug Target Module** | **PI3K-AKT Module** | **Cell Death Module** | **Immune Activation Status Module** | **Pan-Tumor Module** | **Immune Cell Typing Module** |
| PD-1 | 4-1BB | Phospho-AKT1 (S473) | BAD | CD127 | MART1 | CD45RO |
| CD68 | LAG3 | Phospho-GSK3B (S9) | BCL6 | CD25 | NY-ESO-1 | FOXP3 |
| HLA-DR | OX40L | Phospho-GSK3A (S21)/Phospho-GSK3B (S9) | BCLXL | CD80 | S100B | CD34 |
| Ki-67 | Tim-3 | INPP4B | CD95/Fas | ICOS | Bcl-2 | CD66b |
| Beta-2-microglobulin | VISTA | PLCG1 | GZMA | PD-L2 | EpCAM | FAP-alpha |
| CD11c | ARG1 | Phospho-PRAS40 (T246) | Cleaved Caspase 9 | CD40 | Her2 | CD14 |
| CD20 | B7-H3 | Phospho-Tuberin (T1462) | p53 | CD44 | PTEN | CD163 |
| CD3 | IDO1 | Pan-AKT | PARP | CD27 | ER-alpha |  |
| CD4 | STING | MET | BIM |  | PR |  |
| CD45 | GITR | Phospho-AKT (T308) |  |  |  |  |
| CD56 |  |  |  |  |  |  |
| CD8 |  |  |  |  |  |  |
| CTLA4 |  |  |  |  |  |  |
| GZMB |  |  |  |  |  |  |
| PD-L1 |  |  |  |  |  |  |
| PanCk |  |  |  |  |  |  |
| SMA |  |  |  |  |  |  |
| Fibronectin |  |  |  |  |  |  |
| Controls |  |  |  |  |  |  |
| Rb IgG |  |  |  |  |  |  |
| Ms IgG1 |  |  |  |  |  |  |
| Ms IgG2a |  |  |  |  |  |  |
| Histone H3 |  |  |  |  |  |  |
| S6 |  |  |  |  |  |  |
| GAPDH |  |  |  |  |  |  |

*Note. (A) visualization markers used to distinguish between the tumor cells and immune cell, and (B) DSP panels and antibodies used in the current study.*

**Table S3.** Details of antibodies used in multiplex immunofluorescence staining.

| **N** | **Antibody** | **Type** | **Concentration** | **Manufactured by** | **Clone** |
| --- | --- | --- | --- | --- | --- |
| 1 | VISTA | Anti-Rabbit | 1/800 | Novusbio | BLR035F |
| 2 | PanCK | Anti-Mouse | 46.3 ug/ml | Ventana | AE1/AE3/PCK26 |
| 3 | TTF1 | Anti-Rabbit | 5.7 ug/ml | Ventana | SP141 |
| 4 | CD3 | Anti-Rabbit | 0.4 ug/ml | Ventana | 2GV6 |
| 5 | TMEM119 | Anti-Rabbit | 1/300 | Abcam | Polyclonal |

*Note.* VISTA: V-domain Ig suppressor of T cell activation; PanCK: Pan Cytokeratin; TTF1: Transcription Termination Factor 1;CD3: cluster of differentiation 3; TMEM119: Transmembrane Protein 119.
