# Supplementary material for: Tumor lineage-specific immune response in brain metastatic disease: opportunities for targeted immunotherapy regimen?
Source: Acta Neuropathol Commun. 2023 Apr 15;11:64. doi: 10.1186/s40478-023-01542-9 (PMC10105417; doi:10.1186/s40478-023-01542-9)
Supplement: Supplementary file 2 — Additional file 2. Fig. S1 Heterogeneity of VISTA and IDO1 expression in immunohistochemistry (IHC)-stained brain metastases tissues. A. Expression of IDO1 and VISTA in IHC-stained BCBM. The numbers refers to a number of that sample (total samples = 9). B. Expression of IDO1 and VISTA in IHC-stained BM-LUAD. The numbers refers to a number of that sample (total samples = 11). Fig. S2 Comparison of immune checkpoint expressions in high expressed ROIs and low expressed ROIs of IHC-stained brain metastases tissues. A. Expression of IDO1 and VISTA in IHC-stained BCBM in high expressed - and low expressed ROI. NLC stands for the normalized logged counts. The numbers next to the BCBM, refer to a number of that sample. B. Expression of IDO1 and VISTA in IHC-stained BM-LUAD in high expressed - and low expressed ROI. NLC stands for the normalized logged counts. The numbers next to the BM-LUAD, refer to a number of that sample. [file 40478_2023_1542_MOESM2_ESM.pdf]

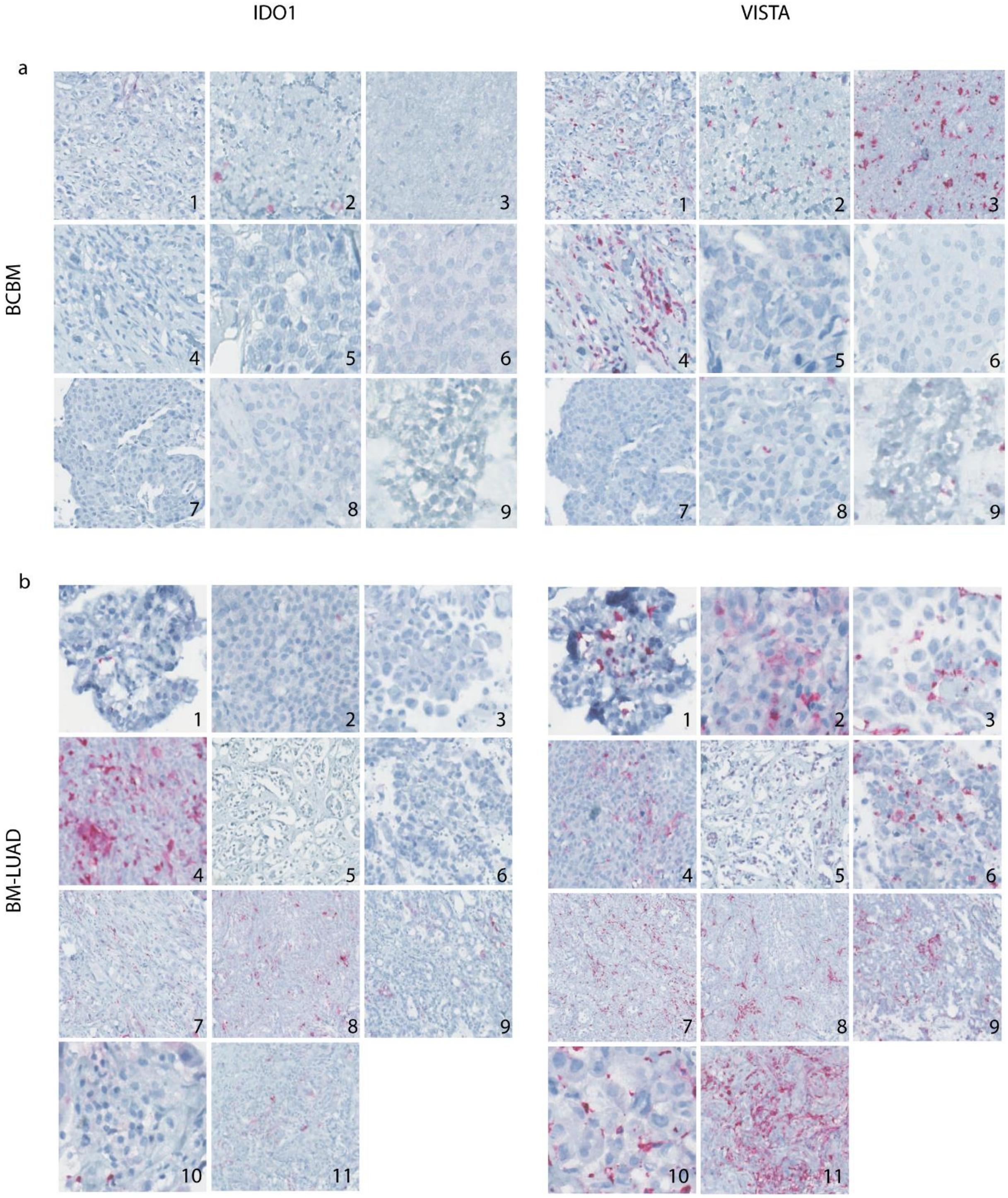

Fig. S1 Heterogeneity of VISTA and IDO1 expression in immunohistochemistry (IHC)-stained brain metastases tissues.

A. Expression of IDO1 and VISTA in IHC-stained BCBM. The numbers refers to a number of that sample (total samples = 9).

B. Expression of IDO1 and VISTA in IHC-stained BM-LUAD. The numbers refers to a number of that sample (total samples = 11).

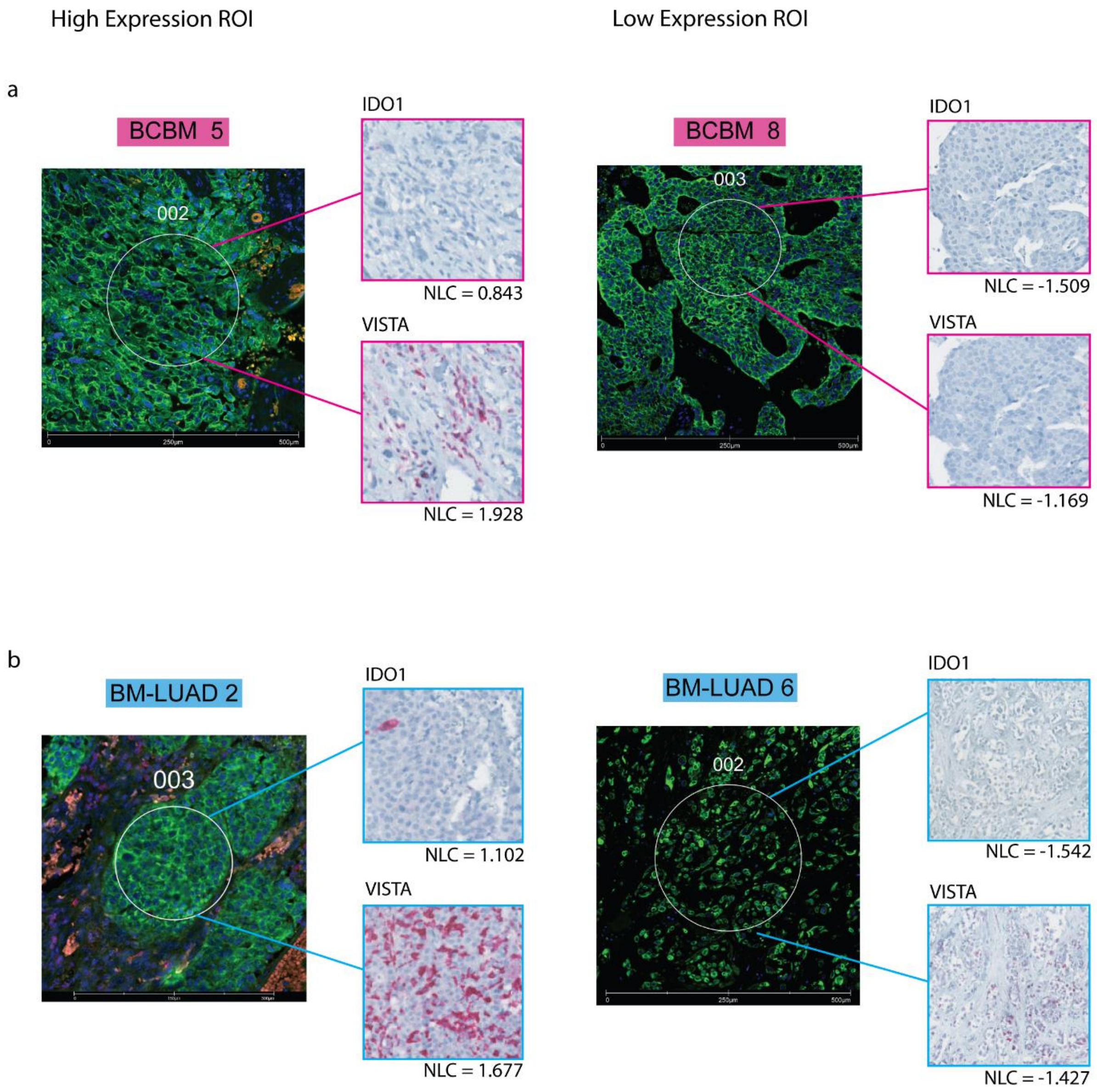

Fig. S2 Comparison of immune checkpoint expressions in high expressed ROIs and low expressed ROIs of IHC-stained brain metastases tissues.

A. Expression of IDO1 and VISTA in IHC-stained BCBM in high expressed - and low expressed ROI. NLC stands for the normalized loged counts. The numbers next to the BCBM, refers to a number of that sample.

B. Expression of IDO1 and VISTA in IHC-stained BM-LUAD in high expressed - and low expressed ROI. NLC stands for the normalized loged counts. The numbers next to the BM-LUAD, refers to a number of that sample.
